# Diagnosis of the cavo-hepato-atrial pathway in Budd-Chiari syndrome by ultrasonography

**DOI:** 10.3892/etm.2014.1828

**Published:** 2014-07-04

**Authors:** YONG-HAO GAI, SHI-FENG CAI, HUI-LI FAN, QING-WEI LIU

**Affiliations:** 1 Department of Ultrasound, Provincial Hospital Affiliated to Shandong University, Jinan, Shandong 250021, P.R. China; 2 Department of Radiology, Provincial Hospital Affiliated to Shandong University, Jinan, Shandong 250021, P.R. China; 3 Department of Ultrasound, Heze Municipal Hospital, Heze, Shandong 274000, P.R. China

**Keywords:** ultrasonography, Doppler, color, hepatic vein thrombosis, collateral circulation

## Abstract

The aim of this study was to investigate the ultrasonic features of the cavo-hepato-atrial pathway in Budd-Chiari syndrome (BCS), by which blood is drained from the occluded inferior vena cava (IVC) to the right atrium via hepatic veins. Ultrasonograms from 11 patients with BCS with cavo-hepato-atrial pathways were retrospectively studied. Doppler ultrasound was used to observe the direction of the flow and measure the velocity of the blood-draining vessels. Blood flow in the draining vessels and the collaterals was shown as blue, red or bicolored depending on whether the flow direction was away from the transducer, towards the transducer or both. For measurement, the Doppler angle between the axis of the Doppler beam and that of the vein examined was always <60°. Ultrasonography was performed 1–2 weeks prior to digital subtraction angiography (DSA). All patients were confirmed by DSA. Membranous and segmental occlusions of IVCs were observed in seven and four cases, respectively. Blood flow from the IVC reversed to the hepatic/accessory hepatic vein, continued through the dilated intrahepatic collaterals, onward to the other hepatic vein and finally to the right atrium. The majority of the inlets (8/11) of hepatic veins above the occlusion were narrow compared with the dilated distant parts of the lumens. Accelerated blood flow in the inlets was detected in all patients regardless of the luminal diameter. In conclusion, the results from the present study suggest that the unusual cavo-hepato-atrial pathway can be diagnosed reliably by ultrasonography, which may be useful for clinical management.

## Introduction

Budd-Chiari syndrome (BCS) is a diverse group of conditions associated with obstruction of the hepatic vein or inferior vena cava (IVC) within or above the liver. In western countries, BCS is often caused by prothrombotic disorders; whilst membranous or segmental obstruction of the IVC is the most common cause of BCS in Asia (1–4). Following obstruction of the IVC, collateral circulation may be developed via azygous, lumbothoracic, intercostal, inferior phrenic and abdominal veins or the portal venous system (5,6). However, blood within the occluded IVC may also be drained to the right atrium by another route, the cavo-hepato-atrial pathway. In the present study, the ultrasonic features of the unusual blood-draining pathway were investigated.

## Materials and methods

### Patients

This study was approved by the Ethics Committee of Shandong Provincial Hospital of Shandong University (Jinan, China). Informed consent was obtained from each patient prior to digital subtraction angiography (DSA), and the protocol was in accordance with the Declaration of Helsinki.

This retrospective study is based on the integrated data of each patient. A total of 11 patients with BCS with IVC obstruction and cavo-hepato-atrial pathways underwent ultrasonic examinations between August 2004 and June 2013. This group of patients comprised 7 males and 4 females aged between 35–73 years (mean age, 49.82±12.15 years). All patients had chronic BCS and the period from first clinical symptoms to diagnosis ranged between 5 months and 10 years. However, a number patients had already suffered from the disease for several years without clear clinical symptoms prior to seeing a doctor; therefore, the duration of the disease could not be determined prior to ultrasonic examination. All patients had primary BCS.

Three patients demonstrated symptoms of right upper abdominal distention, and one patient had decreased appetite. The remaining patients showed no overt symptoms and underwent checkups. Physical examinations revealed hepatomegaly in five patients and no evident signs of ascites, leg edema or superficial venous dilatation in all patients. The laboratory tests revealed that the liver functions of the patients were within the normal range.

### Ultrasonic examination

Ultrasonography was performed using Logiq E9 (GE Healthcare, Vienna, Austria) and Envisor HD (Philips Healthcare, Andover, MA, USA) with multi-frequency convex transducers (3–5 MHz). All patients fasted for >8 h prior to examination. First, the liver, the spleen and the IVC were observed in order to investigate whether hepatomegaly, splenomegaly, expansion of the portal vein and ascites were present. In the presence of hepatic vein or IVC obstruction, the afflictions and the blood-draining pathways were observed and recorded. Doppler ultrasound was used to observe the direction of the flow and measure the velocity of the blood-draining vessels. Blood flow in the draining vessels and the collaterals was shown as blue, red or bicolored depending on flow direction away from the transducer, towards the transducer or both. For measurement, the Doppler angle between the axis of the Doppler beam and that of the vein examined was always <60°. Ultrasonography was performed 1–2 weeks prior to DSA. All patients were confirmed by DSA.

## Results

**Figure 1:**
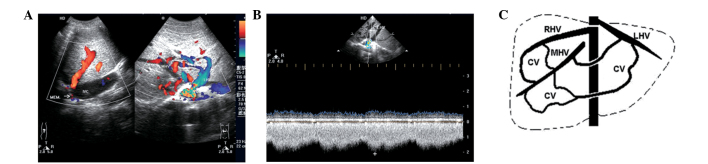
Cavo-hepato-atrial pathway in a 38-year-old male. (A) Membranous occlusion was observed above the RHV, and blood flow from the IVC reversed to the RHV (on the left side), continued through the dilated intrahepatic collaterals, onward to the LHV (orifice above the membrane), the IVC and finally to the right atrium (on the right side). The inlet of the LHV was narrow and showed the aliasing effect. The proximal part of the MHV was occluded and blood inside was drained to the LHV through the intrahepatic collaterals. Hepatomegaly was revealed. (B) Doppler spectrum at the inlet detected at a high flow velocity of 213 cm/sec, with pressure gradient of 18.2 mmHg. (C) Diagram showing the cavo-hepato-atrial pathway. RHV, right hepatic vein; IVC, inferior vena cava; LHV, left hepatic vein; MHV, middle hepatic vein.

**Figure 2:**
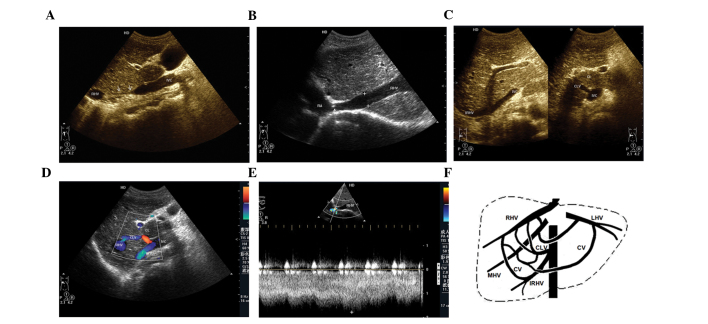
Cavo-hepato-atrial pathway in a 73-year-old male. (A) Segmental occlusion of the IVC. (B) Dilated RHV (diameter, 1.78 cm) with membrane-stenosis inlet (arrow). (C) Dilated IRHV and CLV. (D) Blood of the IVC retrogradely flows to the MHV via the CLV and IRHV. (E) Doppler spectrum at the inlet of the MHV to the right atrium detected at a high flow velocity of 180 cm/sec, with pressure gradient of 12.9 mmHg. (F) Diagram showing the cavo-hepato-atrial pathway. RHV, right hepatic vein; IVC, inferior vena cava; IRHV, inferior right hepatic vein; CLV, caudate lobe vein; MHV, middle hepatic vein; LHV, left hepatic vein.

The ability to diagnose the cavo-hepato-atrial pathway, an unusual collateral circulation with specific hemodynamics in BCS, by ultrasonic examination was evaluated in the present study. Ultrasonography was performed in 11 patients and the results were retrospectively analyzed. Membranous and segmental occlusions of IVCs were detected in seven and four cases, respectively, and occluded hepatic veins were identified in all patients with the exception of the draining hepatic or accessory hepatic veins (including inferior hepatic and caudate veins) that communicate with the IVC and the right atrium. The inlets of eight hepatic veins, which drain to the right atrium, were found to be narrow compared with the dilated distant parts of the lumens. The narrowness was primarily caused by the membrane surrounding the inlets. Blood flow from the IVC reversed to the hepatic/accessory hepatic vein (orifice below occlusion) and then continued through the dilated intrahepatic collaterals, onward to the other hepatic vein (orifice above occlusion), and finally to the right atrium. Accelerated blood flow in the inlets of draining hepatic veins to the right atrium was detected in all patients regardless of the luminal diameter of the inlets. All patients were associated with at least one obstructed hepatic vein, and blood flowing to the draining veins (Figs. 1 and 2; Table I). Minimal dilated azygous and hemiazygous veins were only identified in 1 patient by DSA. These results indicate that the cavo-hepato-atrial pathway is an unusual collateral circulation with specific hemodynamics in BCS, which may be diagnosed by ultrasonic examination.

## Discussion

The cavo-hepato-atrial pathway is an unusual blood-draining pathway of BCS, which results from IVC obstruction. Compared with other collateral circulations (5,6), this pathway is a direct route of drainage from the IVC to the right atrium (7). The pathological change is that blood pressure below the obstructed portion of the IVC exceeds that of the hepatic veins. The continuously increasing pressure in the IVC produces small anastomoses between adjacent intrahepatic veins, which eventually develop into enlarged collaterals (5). Therefore, the hemodynamics of the IVC and hepatic/accessory hepatic veins change accordingly. Blood from the IVC retrogradely flows to the hepatic or accessory hepatic veins (orifice below occlusion), through the enlarged intrahepatic collaterals and the draining hepatic veins (orifice above occlusion), and finally to the right atrium. This blood-draining mechanism relieves the symptoms caused by portal hypertension and IVC hypertension (8,9), and is also why no other collateral circulations were identified in the majority of the patients investigated in the present study. In addition, as shown in the literature, this blood-draining mode efficiently compensates the IVC outflow to the heart, and treatment of the obstruction of the IVC may be deemed unnecessary for patients (9). Although the authors of the present study are amenable to this view, they consider that it may be necessary for certain patients to undergo angioplasty. When short segmental (15 mm< occluded length ≤20 mm) or membranous occlusions of the IVC exist and are easily managed by interventional therapy, re-canalization of the IVC may bring the hemodynamics back to normal and efficiently relieve the hypertension of the IVC, the hepatic veins or accessory hepatic veins. Although the inlets above the obstruction in certain patients were relatively narrow, blood may be efficiently drained into the IVC by other hepatic veins or accessory hepatic veins with inlets below the obstruction following the surgery. Eight patients with membranous or short segmental occlusion of IVC in the present study underwent angioplasty for re-canalization of the IVC, and the blood direction returned to normal. In addition, the velocity of the inlets of the draining veins to the right atrium markedly decreased. For patients with a long segmental occlusion (occluded length >20 mm) of the IVC that is difficult to manage, follow-up is required. The remaining three patients in the present study were followed up for 1–5 years without surgery, and their ultrasonograms and clinical manifestations did not change significantly. The membrane that results in relative narrowness of the inlet may be part of the IVC wall, which is derived from the IVC-wall-limited dilatation of the inlet rather than the clearly expanded distant lumen of the draining vein.

**Figure 3:**
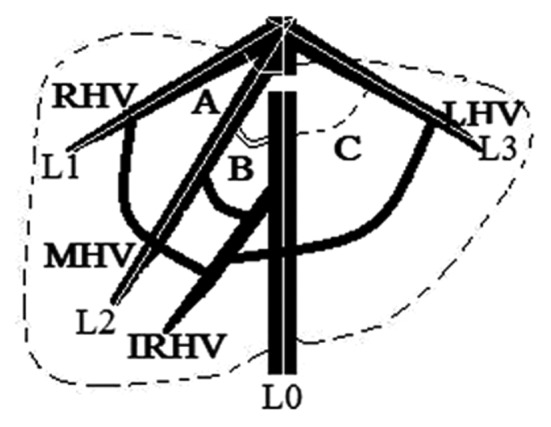
Diagram showing the angles between long axes of the blood flow of the RHV (A, white arc), MHV (B, double black arcs), LHV (C, dashed black arc) and IVC, respectively. The white lines L0 to L3 indicate the long axes of the blood flow of the IVC, RHV, MHV and LHV, respectively. RHV, right hepatic vein; IVC, inferior vena cava; LHV, left hepatic vein; MHV, middle hepatic vein; IRHV, inferior right hepatic vein.

With the help of hemodynamics, ultrasonic examination provides a convenient and accurate method for the diagnosis of the rare pathway. The diagnosis is based on the following conditions: i) obstruction of the IVC; ii) one hepatic vein above the obstruction and another hepatic vein or accessory hepatic vein below the obstruction are open to the IVC (in certain cases the former hepatic vein is open directly to the right atrium); iii) blood flow from the IVC reverses to the hepatic or accessory hepatic vein, the intrahepatic collaterals, and the other draining hepatic vein above the obstruction, and then flows into the right atrium, with the intrahepatic communicating branches receiving blood from the obstructed hepatic veins; and iv) angles between the long axis of blood flow and the IVC may be observed due to the existence of angulation between the long axis of the hepatic vein and the IVC (Fig. 3).

In a previous study, we determined a number of ultrasonic features of draining pathways in BCS (10). In the present study, further investigation of the features of the cavo-hepato-atrial pathway was conducted, which provided additional information for the diagnosis of this rare pathway. In conclusion, the cavo-hepato-atrial pathway is an unusual collateral circulation with specific hemodynamics in BCS. Ultrasonic examination provides an accurate method for the diagnosis of this rare pathway.

## Figures and Tables

**Table I:** Ultrasonographic descriptions of Budd-Chiari syndrome with cavo-hepato-atrial pathways.

				Draining vein to the right atrium		
				
Patient no.	Age (years)/gender (M/F)	Occlusion of IVC	Draining vein(s) from IVC	Description	Status of inlet	Velocity of inlet (cm/sec)
1	73/M	Segmental	IRHV and CLV	RHV	Narrowing	180
2	59/F	Membranous	IRHV and CLV	LHV	Narrowing	115
3	35/M	Membranous	RHV	MHV	Narrowing	131
4	58/M	Segmental	CLV	RHV	Patent	63
5	44/M	Membranous	MHV	RHV	Narrowing	154
6	51/M	Segmental	IRHV	MHV	Narrowing	105
7	47/F	Membranous	RHV	MHV	Narrowing	135
8	38/M	Membranous	RHV	LHV	Narrowing	213
9	43/F	Membranous	IRHV	MHV	Patent	87
10	63/M	Membranous	IRHV and CLV	LHV	Patent	74
11	37/F	Segmental	IRHV	MHV	Narrowing	144

Segmental occlusion of IVC: occluded length >1.5 cm; membranous occlusion of IVC: membrane thickness ≤1.5 cm. IVC, inferior vena cava; RHV, right hepatic vein; MHV, middle hepatic vein; LHV, left hepatic vein; IRHV, inferior right hepatic vein; CLV, caudate lobe vein.
